# Two-Dimensional Layered Nanomaterial-Based Electrochemical Biosensors for Detecting Microbial Toxins

**DOI:** 10.3390/toxins12010020

**Published:** 2019-12-31

**Authors:** Zhuheng Li, Xiaotong Li, Minghong Jian, Girma Selale Geleta, Zhenxin Wang

**Affiliations:** 1 Jilin Provincial Institute of Education, Changchun 130022, China; lizhuheng@126.com; 2 State Key Laboratory of Electroanalytical Chemistry, Changchun Institute of Applied Chemistry, Chinese Academy of Science, Changchun 130022, China; lixiaotong@ciac.ac.cn (X.L.); mhjian@ciac.ac.cn (M.J.); 3 Department of Chemistry, College of Natural Sciences, Jimma University, Jimma 378, Ethiopia

**Keywords:** two dimensional layered nanomaterials, electrochemical biosensors, microbial toxin detection, antibodies, aptamers

## Abstract

Toxin detection is an important issue in numerous fields, such as agriculture/food safety, environmental monitoring, and homeland security. During the past two decades, nanotechnology has been extensively used to develop various biosensors for achieving fast, sensitive, selective and on-site analysis of toxins. In particular, the two dimensional layered (2D) nanomaterials (such as graphene and transition metal dichalcogenides (TMDs)) and their nanocomposites have been employed as label and/or biosensing transducers to construct electrochemical biosensors for cost-effective detection of toxins with high sensitivity and specificity. This is because the 2D nanomaterials have good electrical conductivity and a large surface area with plenty of active groups for conjugating 2D nanomaterials with the antibodies and/or aptamers of the targeted toxins. Herein, we summarize recent developments in the application of 2D nanomaterial-based electrochemical biosensors for detecting toxins with a particular focus on microbial toxins including bacterial toxins, fungal toxins and algal toxins. The integration of 2D nanomaterials with some existing antibody/aptamer technologies into electrochemical biosensors has led to an unprecedented impact on improving the assaying performance of microbial toxins, and has shown great promise in public health and environmental protection.

## 1. Introduction

Two-dimensional layered (2D) nanomaterial (e.g., graphene and its derivatives, transition metal dichalcogenides (TMDs) and other layered nanosheets)-based electrochemical signal amplifications have great potential for improving both the sensitivity and selectivity of electrochemical biosensors because of their unique physical, chemical, and electrical properties [1,2,3,4,5,6,7,8,9,10,11,12,13,14,15,16,17,18,19,20,21]. Graphene is a single layer of densely packed carbon atoms with a benzene-ring structure, and is the first known 2D layered nanomaterial [22]. The unique properties of graphene, including its exceptional mechanical strength [23], extremely large surface area (2630 m^2^/g) [24], very high thermal conductivity in the range of ∼3080–5150 W mK^−1^ [25], high conductivity [26], good charge carrier mobility [27], and wide potential window [28], endow it with great applicability in the development of biosensors, and, in particular, electrochemical biosensors [1,2,3,4,5,12,22,23,24,29]. In addition, based on the molar ratio of carbon to oxygen (C/O), graphene can be roughly divided into two categories, graphene oxide (GO) or reduced graphene oxide (rGO). It is demonstrated that rGO has better electrical conductivity than GO. Because pure graphene lacks an intrinsic band gap and is limited by chemical modification, there is an increasing interest in synthesizing graphene derivatives/nanocomposites and graphene-like 2D nanomaterials. Among the graphene-like 2D nanomaterials, TMDs (e.g., molybdenum disulfide (MoS_2_) and molybdenum selenide (MoSe_2_)) show excellent physicochemical properties and remarkable biocompatibility, and also have significant attraction for the fabrication of electrochemical (bio)sensors [6,7,9,14,21,29]. Driven by their unprecedented properties, massive synthetic methods/protocols have been developed for preparing 2D nanomaterials and 2D nanomaterial composites, which involves both physical strategies and chemical approaches, such as dry mechanical exfoliation (e.g., Scotch tape), chemical (e.g., solution-based exfoliation, graphite oxide exfoliation/reduction) and/or electrochemical (oxidation/reduction and exfoliation) processes, chemical vapor deposition (CVD), chemical synthesis, thermal decomposition of SiC wafers and unzipping carbon nanotubes [26,30,31,32,33,34,35,36,37,38,39,40,41,42,43]. In this review, we will not describe the detailed synthetic methods/protocols mentioned above for synthesis of 2D nanomaterials, however, we suggest reading several recently published comprehensive review articles [40,41,42,43,44,45]. These methods of 2D nanomaterial preparation produce different forms of nanomaterials with a diversity of properties including mechanical, optical, electrical, chemical and biological properties. These diverse properties make 2D nanomaterials suitable for an extensive range of applications, such as drug delivery, in vitro and in vivo imaging, tissue engineering, biosensor construction, and energy conversion and storage [39,43,46]. For biosensor applications, 2D nanomaterials should be extensively characterized because their properties strongly dependent on their characteristics such as thickness or number of layers, morphology, chemical structure and surface functional groups.

Microbial toxins are the general term for a class of substances covering a broad range from small molecules to biomacromolecules (e.g., peptides and proteins), which are produced by living organisms including bacteria, fungus and algae [47,48,49,50,51,52,53]. They are widespread throughout the whole world, threatening the health and/or life of humans and livestock, and affecting domestic and international trade. For instance, aflatoxin B1 (AFB1, a kind of mycotoxin produced by fungi) has been defined as a group I carcinogen by the World Health Organization (WHO) [53]. Some microbial toxins can generate acute poisonous effects even at very low doses, and the co-occurrence of microbial toxins in nature may cause significantly additive and/or synergistic toxicity. In order to efficiently avoid potential hazards on public health and safety, it is important to precisely and reliably determine the toxins in practical samples from different sources. Liquid chromatography-based methods including high-performance liquid chromatography (HPLC) and high-performance liquid chromatography-tandem mass spectrometry (HPLC/MS/MS) are the gold standards for accurate analysis of toxins [54,55,56,57,58,59]. Although the HPLC-based methods have high reliability and accuracy, they typically require expensive laboratory facilities and instruments, complex pre-treatment processing of the sample and well-trained operators. These drawbacks strongly limit the application of HPLC-based methods in on-site detections of toxin. Various sensing systems such as surface plasmon resonance (SPR) biosensors, electrochemical biosensors, fluorescence biosensors, colorimetric assays, competitive enzyme-linked immunosorbent assay (ELISAs) and microfluidic immunoassay have been developed for analysis of toxins from different sources including clinical samples, foods, water and feeds [60,61,62,63,64,65,66]. Among these biosensing systems, electrochemical biosensors and biotransducers are more attractive because they offer several advantages such as high sensitivity, operational simplicity, relatively low cost, easily miniaturization and suitable on-site analysis [8,11,12,13,14,15,16,17,18,19,67,68,69]. These advantages make electrochemical biosensors/transducers of microbial toxins powerful tools in many areas including food, environmental and medical monitoring, disease diagnosis and anti-terrorism security. Owing to the large surface areas and excellent conductivities, the integration of 2D nanomaterials (e.g., graphene and TMDs) and their nanocomposites with electrochemical transducers has great potential to enhance the analytical performance of electrochemical biosensors for detection of toxins [8,11,12,13,14,15,16,17,18,19]. For example, since its birth, multiple research initiatives on graphene applied to electroanalytical chemistry have been launched worldwide, and analysts have been developing a plethora of different graphene-based electrochemical sensing platforms for detection of various targets including microbial toxins. Typically, these electrochemical biosensors comprise a graphene and/or a graphene derivative/nanocomposite-modified electrode as an electrochemical signal transduction element, and a biological recognition element (e.g., antibodies, aptamer and microbial cells). The signal from the biological recognition event is converted to a quantifiable electrical signal because the biological target is normally in close contact with the electrochemical signal transduction element through physical or chemical interactions (e.g., electrostatic interactions, π-π interactions and covalent bonds). Because of their unique properties (e.g., large surface area and good conductivity), the detection performance of an electrochemical biosensor can be significantly improved by using the graphene and/or a graphene derivative/nanocomposite. Therefore, the scope of application of 2D nanomaterial-based electrochemical biosensors has been constantly expanding in the field of toxin detection. Some of these studies have been reviewed elsewhere with a focus on the fabrication and toxin detection of graphene-based electrochemical biosensors or as subclassifications in more generalized overviews of the nanomaterial-based electrochemical biosensors [8,11,12,13,14,15,16,17,18,19]. In this review, we will focus on the recent development of GO/rGO and/or MoS_2_/MoSe_2_-based electrochemical biosensors for the determination of various microbial toxins, such as bacterial toxins, fungal toxins and algal toxins, highlighting some of their current achievements, technical challenges/limitations and the future directions by means of a set of selected recent publications.

## 2. Detection of Bacterial Toxins

### 2.1. Botulinum Neurotoxins

**Figure 1:**
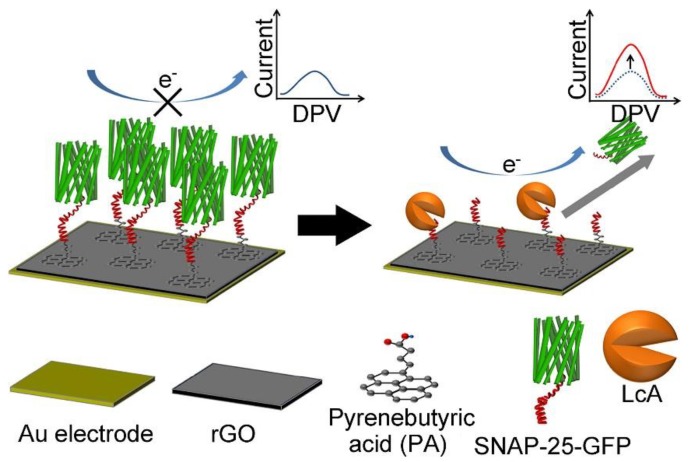
Schematic representation of the detection principle of the rGO based electrochemical biosensors (adapted from Chan et al. 2015 [75], Copyright 2015 Elsevier B.V. and reproduced with permission).

The *Botulinum* neurotoxins (BoNTs), which are produced by *Clostridium botulinum*, an anaerobic bacterium, are among the most toxic of all naturally occurring substances [70,71,72]. Based on their molecular structures, BoNTs are categorized into seven serotypes (from A to G). They inhibit acetylcholine release from presynaptic nerve terminals at the neuro-muscular junction in both the central and peripheral nervous systems through cleavage of soluble N-ethylmaleimide-sensitive factor attachment protein receptors (SNAREs), resulting in flaccid muscle paralysis. BoNTs can cause the deadly disease, botulism, with a median lethal dose (LD50) of 1 ng per kg bodyweight. Foods are easily contaminated by *Clostridium botulinum* during processing. Various (impedimetric, voltammetric and amperometric) electrochemical biosensors have been fabricated for BoNT detection [73,74,75,76]. In particular, electrochemical biosensors can achieve detection of this toxin in a fast and meticulous way, and they also provide a robust and cost-effective approach for real-time monitoring of BoNTs. Recently, 2D nanomaterial-based electrochemical biosensors have been applied to sensitively detect BoNTs in various samples including foods. For instance, Narayanan et al. constructed an electrochemical immunosensor of the BoNT serotype E (BoNT/E) by using graphene nanosheets–aryldiazonium salts as transducers [74]. The as-proposed immunosensor shows a low limit of detection (LOD, 5 pg mL^−1^) and can be employed for rapid detection of BoNT/E with a total analysis time of 65 min. Chan et al. fabricated an electrochemical biosensor for ultrasensitive detection of BoNT serotype A light chain (BoNT-LcA) through immobilization of the SNAP-25-GFP (synaptosomal associated protein 25-green fluorescent protein) peptide substrate on the rGO modified gold electrode via a pyrenebutyric acid (PA) linker (as shown in Figure 1) [75]. In this case, PA was immobilized on the rGO surface through π-π stacking. Subsequently, SNAP-25-GFP peptide reacted with PA via N-(3-dimethylaminopropyl)-N’-ethylcarbodiimide hydrochloride/N-hydroxysulfosuccinimide (EDC/Sulfo-NHS) activation. After specific cleavage of SNAP-25-GFP by BoNT-LcA, the steric hindrance and electrostatic repulsion of SNAP-25-GFP decreased, resulting in an increase in the electrochemical signal. The amount of BoNT-LcA can be detected through the change of peak current of the electrochemical redox probe (ferricyanide, [Fe(CN)_6_]^3−/4−^(1:1)) by the differential pulse voltammetry (DPV) measurement. The as-fabricated electrochemical biosensor provides a relatively wide linear range (1 pg mL^−1^ to 1 ng mL^−1^) and a relatively low LOD (8.6 pg mL^−1^) for detection of BoNT-LcA because the rGO modified Au (rGO/Au) electrode provides a robust and biocompatible platform with improved electron transfer capability and a large surface area for peptide immobilization. The feasibility of the as-fabricated biosensor is demonstrated by detection of BoNT-LcA in spiked milk samples. Afkhami et al. developed a gold nanoparticle-graphene-chitosan (Au NPs-Gr-Cs) nanocomposite-based impedimetric immunosensor for the detection of BoNT serotype A (BoNT/A) [76]. The Au NPs-Gr-Cs nanocomposite was used for the amplification of the electrochemical signal, and monoclonal anti-BoNT/A antibodies were conjugated on the Au NPs-Gr-Cs nanocomposite modified glassy carbon electrode (GCE). In the presence of BoNT/A, the immunocomplex formed on the as-prepared electrode surface, which acts as the inert electron and mass transfer blocking layer. Therefore, the diffusion of [Fe(CN)_6_]^3−/4−^ is hindered, resulting in a decrease of the peak current. The Au NPs-Gr-Cs nanocomposite-based impedimetric immunosensor has an excellent linear range (from 0.27 to 268 pg mL^−1^) with a LOD of 0.11 pg mL^−1^, and is very suitable for routine analysis of BoNT/A in different matrices, such as serum and milk.

### 2.2. Clostridium difficile Toxin B

**Figure 2:**
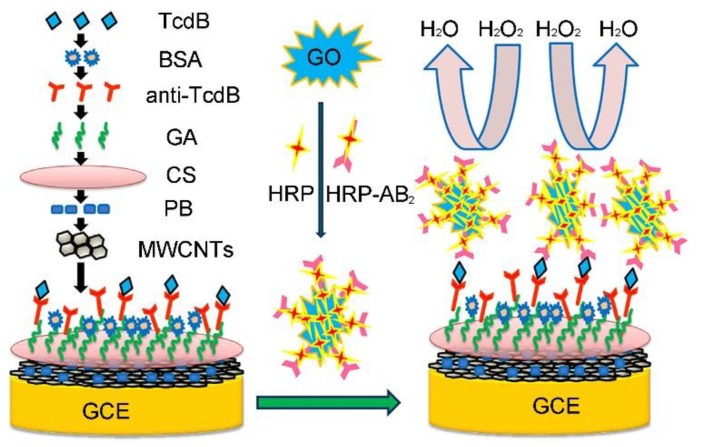
Schematic representation of the immunosensor array preparation and detection strategy by sandwich-type immunoassay of Tcd B. Here, Tcd B means *C. difficile* toxin B, BSA means bovine serum albumin, anti-Tcd B means anti-Tcd B antibody, HRP means horseradish peroxidase, HRP-Ab2 means HRP-labeled second anti-Tcd B antibody, GA means glutaraldehyde, CS means chitosan, PB means Prussian blue, MWCNTs means multi-walled carbon nanotube, GO means graphene oxide, and GCE means glassy carbon electrode (adapted from Fang et al. 2014 [84], Copyright 2013 Elsevier B.V. and reproduced with permission).

*Clostridium difficile* toxin A (Tcd A, 308 kDa) and toxin B (Tcd B, 270 kDa) are co-produced by *Clostridium difficile (C. difficile)*. Tcd A is an enterotoxin responsible for tissue damage, while Tcd B is referred to as a potent cytotoxin [77,78,79,80,81]. In particular, the rapid and sensitive detection of Tcd B is very helpful for early diagnosis and efficient therapy because Tcd B is critical for virulence and is found in all clinically isolated pathogenic strains [79,80,81,82,83,84,85]. Using the advantages of GO, including the large surface area and good conductivity, Fang et al. developed a simple sandwich-assay type electrochemical immunosensor for improving the Tcd B detection sensitivity by using GO as a scaffold for the enhanced loading of horseradish peroxidase (HRP) and HRP-labeled secondary Tcd B antibody (as shown in Figure 2) [84]. The LOD (0.7 pg mL^−1^) of the sandwich-assay type electrochemical immunosensor is much lower than those of other current techniques including ELISA. In addition, the as-prepared electrochemical immunosensor was successfully employed to detect Tcd B in practical samples (e.g., real human stool), demonstrating that the immunosensor has promising potential in clinical applications.

### 2.3. Staphylococcal Enterotoxin B

**Figure 3:**
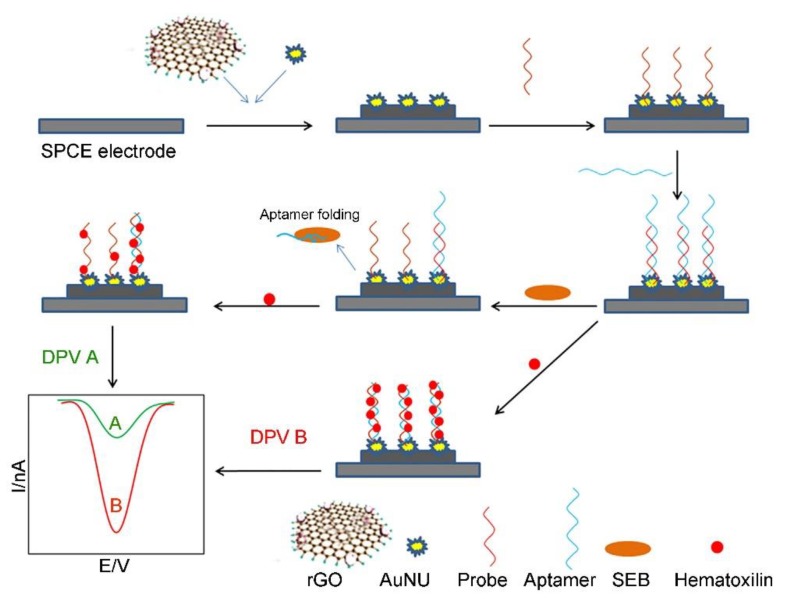
Schematic representation of the fabrication process of the SEB aptasensor by using rGO and AuNU-modified screen printed carbon electrodes (SPCEs) (adapted from Nodoushan et al. 2019 [91], Copyright 2018 Elsevier B.V. and reproduced with permission).

Among the toxins secreted by *Staphylococcus aureus*, the staphylococcal enterotoxin B (SEB) shows superantigenic properties in nature. SEB exposure can result in immunosuppression and serious food poisoning [86,87]. Therefore, it is important to develop a cost-effective, easy-to-use, rapid and sensitive method for real-time monitoring of a low concentration (less than 20 ng kg^−1^ (i.e., LD_50_ value)) of SEB in foods. Several graphene-based electrochemical biosensors have been developed for real-time detection of SEB in foods with a high sensitivity [88,89,90,91]. For instance, Sharma et al. reported on an electrochemical biosensor based on a rGO-chitosan-AuNPs-capturing antibody (rGR-Ch-AuNPs-CAb)-modified GCE for detecting SEB [88]. The rGR-Ch-AuNPs-CAb modified GCE shows remarkable detecting performance because it has a flat two-dimensional configuration and large surface area with plenty of active sites (i.e., functional groups). Using the as-proposed rGR-Ch-AuNPs-CAb-based electrochemical biosensor, 5 ng mL^−1^ SEB can be easily detected within 35 min, which is much lower than the LD_50_ value of SEB. Very recently, Nodoushan et al. fabricated an electrochemical aptasensor for SEB detection by using a rGO and gold nano-urchins (AuNUs)-modified screen printed carbon electrode (SPCE) (as shown in Figure 3) [91]. The aptamer of SEB was attached on the electrode surface through hybridization with the immobilized single-stranded DNA probe on the surface of the AuNUs. Hematoxylin was used as the electrochemical signal generator. In the presence of SEB, the aptamer released from the electrode surface, resulting in an increase in the peak current of hematoxylin. Benefiting from the high conductivity of rGO and high surface area of AuNUs, a wide linear range from 5.0 to 500.0 fmol L^−1^ was achieved and the LOD was calculated as 0.21 fmol L^−1^. There is no significant difference between the results given by the commercial ELISA kit and the electrochemical aptasensor. In particular, the aptasensor shows better recovery rates and lower standard deviation than those of the commercial ELISA kit, which could be employed as a point-of-care (POC) device for assessing food samples.

## 3. Detection of Fungal Toxins

### 3.1. Aflatoxins

**Figure 4:**
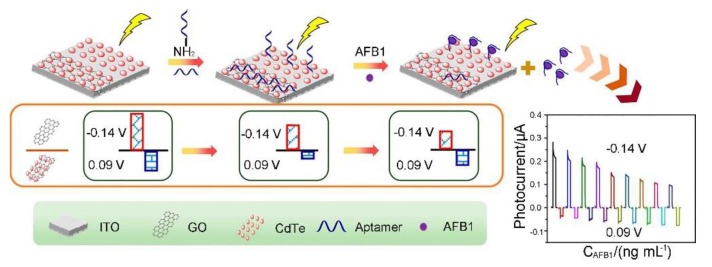
Schematic representation of the construction of the self-reference photoelectrochemical (PEC) biosensor for the detection of AFB_1_ (adapted from Hao et al. 2017 [104], Copyright 2017 American Chemical Society and reproduced with permission).

Aflatoxins are a widespread group of food toxins that are produced by *Aspergillus flavus* and *Aspergillus parasiticus* [92,93,94,95]. There are four main types of aflatoxins: B_1_, B_2_, G_1_, and G_2_, which are based on their fluorescence characteristics under UV light (blue or green) excitation and relative chromatographic mobility in thin-layer chromatography. Among the aflatoxins, AFB1 is considered the most toxic aflatoxin, and can cause cancers, such as hepatocellular carcinoma. Various 2D nanomaterial-based electrochemical biosensors have been constructed for detecting AFB_1_ in various matrixes [96,97,98,99,100,101,102,103,104,105,106,107,108,109,110,111,112,113]. Srivastava et al. have developed a series of functionalized GO nanocomposite-based electrochemical biosensors for profiling AFB_1_ in foods since they developed the first rGO-based AFB_1_ immunosensor through the covalent conjugation of the monoclonal anti-AFB1 antibodies onto an rGO modified indium tin oxide (ITO) electrode in 2013 [96,97,98,99]. Among these electrochemical biosensors, the functionalized GO/rGO-based nanocomposites are employed in different roles, such as catalysts, electroactive probes and immobilization platforms for improving the biosensing performance. For instance, benefiting from the highly crystalline properties of the rGO-Ni NPs sheets (Ni nanoparticle decorated rGO sheets) along with the excellent electro-catalytic properties, the rGO-Ni NPs-ITO-based AFB_1_ immunosensor exhibits high sensitivity (129.6 mA ng^−1^ mL cm^−2^), long term stability (up to 6 weeks) and low LOD (0.16 ng mL^−1^) [99]. Photoelectrochemical (PEC) biosensors have attracted great attention in the biological analytical field as the PEC method can obtain high sensitivity without expensive equipment. Recently, Hao et al. developed a dual channel self-reference PEC biosensor for detecting AFB_1_ through immobilization of the AFB_1_ aptamer onto cadmium telluride (CdTe) and the CdTe-GO modified ITO electrode (as shown in Figure 4) [104]. In this case, CdTe and CdTe-GO were used to generate an anodic photocurrent and cathodic photocurrent, respectively. The AFB1 aptamer was immobilized on the PEC active materials, CdTe and CdTe-GO, through a covalent reaction or physical absorption, respectively. In the presence of AFB1, the aptamer is released from the CdTe-GO surface, resulting in the recovery of the cathodic photocurrent, while the aptamer forms an aptamer-AFB1 complex on the CdTe surface, and the anodic photocurrent decreases further. Compared to traditional PEC biosensors, the CdTe/CdTe-GO-based dual channel self-reference PEC biosensor can provide better precision and reliability, which is promising for detection of AFB_1_ in complex matrixes. Very recently, Peng et al. developed an AFB1 electrochemical aptasensor based on tetrahedral DNA nanostructures (TDNs) immobilized on three dimensionally ordered macroporous MoS_2_-AuNPs hybrids (3DOM MoS_2_-AuNPs) [107]. 3DOM MoS_2_-AuNPs can enhance the immobilization amount of TDNs and facilitate the movement of the electrons between the electrode surface and the redox probe. In combination with a HRP functionalized magnetic signal amplifier, the aptasensor achieves a good linear range (from 0.1 fg mL^−1^ to 0.1 μg mL^−1^) and a LOD of 0.01 fg mL^−1^, which can be employed to detect AFB_1_ in grain products such as rice and wheat powder samples.

### 3.2. Ochratoxin

**Figure 5:**
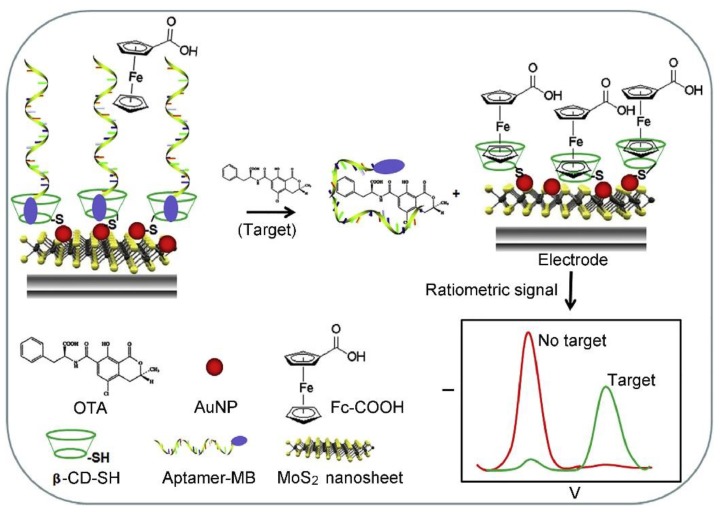
Schematic representation of the fabrication of the ratiometric electrochemical aptasensor for OTA detection based on nanocomposites of gold nanoparticle and MoS_2_ nanosheets with β-CD-SH (thiolated β-CD) (adapted from Wang et al. 2018 [128], Copyright 2018 Elsevier Ltd. and reproduced with permission).

Ochratoxin A (OTA) is the major mycotoxin of the ochratoxin group, which are produced primarily by fungi (e.g., *Aspergillus ochraceus, Penicillium verrucosum* and *Aspergillus niger*) [114,115,116,117]. OTA has strong nephrotoxicity, and is the main etiological agent responsible for human Balkan endemic nephropathy (BEN) and associated urinary tract tumors. In addition, high concentrations of OTA has certain hepatotoxicity. During the last 5 years, several 2D nanomaterial-based electrochemical biosensors including immunosensors and aptasensors have also been developed for sensing OTA [118,119,120,121,122,123,124,125,126,127,128,129,130,131]. For instance, a series of aptasensors based on rGO-AuNP nanocomposites have been constructed by Wang’s group [118,119,120]. The rGO-AuNP nanocomposites have well-dispersity and controllable surface coverage of AuNPs on the rGO sheet, which can be employed as an excellent signal amplified platform for an impedimetric aptasensor and/or an efficient nanocarrier for the CdTe QD (cadmium telluride quantum dot)-based amperometric aptasensor. As a typical example, a label free electrochemical aptasensor was successfully fabricated for ultrasensitive detection of OTA through using the CdTe QDs modified graphene/AuNPs nanocomposite (GAu/CdTe) as a signal amplifier. The as-proposed label-free amperometric aptasensor exhibits a wide linear range from 0.2 pg mL^−1^ to 4 ng mL^−1^ and a low LOD (0.07 pg mL^−1^), which has great potential in various applications, such as food safety monitoring and clinical diagnosis [120]. Bulbul et al. developed a non-enzymatic nanocatalyst-based amperometric aptasensor for OTA detection through immobilization of the OTA aptamer on the GO-modified electrode and the electro-oxidation of a nanoceria (nCe) tag [121]. In this case, GO was used as an electrode material for facilitating the electron transport and enhancing the electrochemical response because it has high conductivity and peroxidase-like activity. In particular, the synergistic effect between the catalase activity of nCe and the peroxidase like activity of GO increases the OTA detection sensitivity significantly. The LOD of as-proposed amperometric aptasensor is calculated to be 0.1 nmol L^−1^, which is below the European Union regulatory limits of OTA (such as 5 μg kg^−^^1^ in raw cereal grains, 3 μg kg^−^^1^ in products derived from cereals, and 2 μg kg^−^^1^ in grape juice). The analytical reliability of the amperometric aptasensor has been demonstrated by the detection of OTA in spiked corn samples. Recently, Wang et al. constructed a ratiometric electrochemical aptasensor for OTA detection through assembly of a methylene blue (MB)-modified OTA aptamer (MB-aptamer) on the MoS_2_ nanosheet/AuNP (MoS_2_-AuNP) nanocomposite-decorated gold electrode through the host-guest recognition of β-cyclodextrin (β-CD) (as shown in Figure 5) [128]. After interaction with OTA, the MB-aptamer was disassembled because of G-quadruplex formation, leading to a decrease in the peak current of MB. Whereas the free ferrocenecarboxylic acid was recognized by β-CD and produced signals in the current, resulting in the “ratiometric” effect. With the combination of high electrocatalytic activity of MoS_2_-AuNP nanocomposites and the recognition capability of β-CD, the as-proposed ratiometric electrochemical aptasensor possesses satisfactory superiority in terms of detection range (from 0.1 nmol L^−1^ to 50 nmol L^−1^), sensitivity (a LOD of 0.06 nmol L^−1^), and accuracy (6.5% of the relative standard deviation (RSD)). The practicability of the aptasensor was successfully demonstrated by detecting OTA in red wine samples.

### 3.3. Mycotoxins Produced by Fusarium

**Figure 6:**
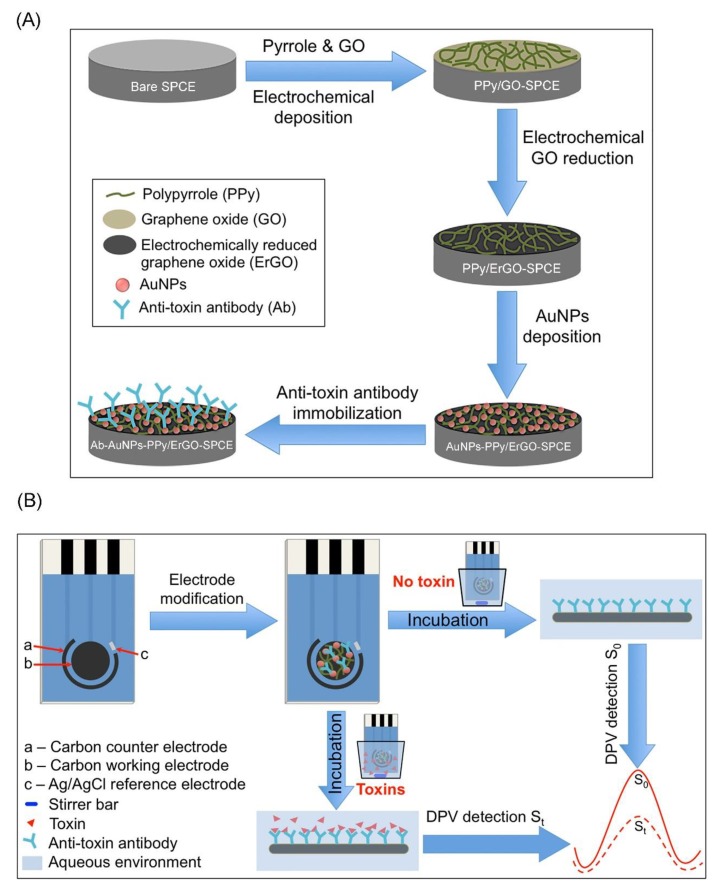
(**A**) Schematic representation of fabrication of the immunosensor and (**B**) detection of mycotoxins (adapted from Lu et al. 2016 [134], Copyright 2016 Elsevier Ltd. and reproduced with permission).

**Figure 7:**
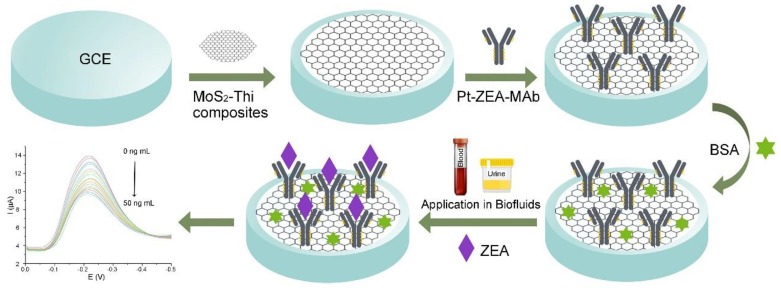
Schematic representation of the electrochemical immunosensor based on MoS_2_-Thi composites for the rapid detection of ZEA in biofluids (adapted from Jiang et al. 2019 [136], Copyright 2019 Elsevier B.V. and reproduced with permission).

The 2D nanomaterial-based electrochemical biosensors have also been developed for detecting other *mycotoxins* produced by *Fusarium* including deoxynivalenol (DON), fumonisin 1 (FB1), and zearalenone (ZEN) [132,133,134,135,136,137]. Shi et al. developed an aptasensor for sensitive FB1 detection by using the dual amplification of AuNPs and graphene/thionine nanocomposites (GSTH) [132]. GSTH served as electrochemical probes, which exhibit a strong electrochemical signal because the graphene has excellent conductivity and a large surface area for immobilizing a large amount of thionine molecules. The as-prepared aptasensor has a six orders of magnitude linear range with a LOD of 1 pg mL^−1^. Lu et al. fabricated an electrochemical immunosensor based on a graphene nanocomposite for rapid and sensitive detection of two mycotoxins, DON and FB1 by using correspondent anti-toxin antibodies (as shown in Figure 6) [134]. In this case, the disposable SPCE was used as a sensing platform, which was modified by AuNPs and polypyrrole (PPy)-electrochemical rGO (PPy/ErGO) nanocomposite film. The film exhibits effective anti-toxin antibody immobilization capacity, enhanced electrical conductivity, and biocompatibility. The current signal of PPy/ErGO-SPCE is much better than that of PPy/rGO-SPCE. Benefiting from the excellent electrochemical response and effective antibody immobilization, the immunosensor exhibits good sensitivity, with a LOD of 4.2 ng mL^−1^ for FB1 and 8.6 ng mL^−1^ for DON. The immunosensor can be used for simultaneous detection of multiple co-contaminant mycotoxins individually in the practical samples (e.g., corn extracts) because it shows low matrix interference even in co-existing toxin environments. Very recently, Jiang et al. constructed a facile electrochemical immunosensor based on thin-layer MoS_2_ and thionin (MoS_2_-Thi) composites for the sensitive and rapid detection of zearalenone (ZEA) in human biofluids (as shown in Figure 7) [136]. The as-prepared MoS_2_-Thi nanocomposites were employed as excellent electrochemical probes, as well as an efficient anti-ZEA antibody loading platform because MoS_2_ retains the electrochemical activity of Thi, and has a large surface area. The MoS_2_-Thi-based electrochemical immunosensor has good ZEA detection performance including a wide linear range (0.01 to 50 ng mL^−1^), low LOD (0.005 ng mL^−1^ ZEA in both the plasma and urine), excellent selectivity, rapid responding time (20 min), acceptable stability (retained more than 85% detection capability at 4 °C for 10 days) and good practicability (detection of ZEA in real human biofluids).

## 4. Detection of Algal Toxins

### 4.1. Microcystins

**Figure 8:**
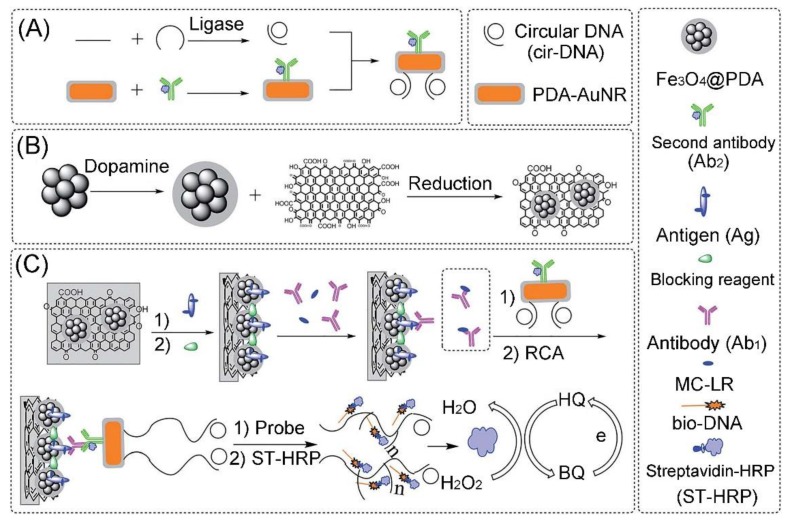
Schematic representation of (**A**) the preparation of Ab_2_-AuNR-cirDNA, (**B**) the formation of magnetic graphene composite, and (**C**) the construction process of the proposed MC-LR immunosensor (adapted from He et al. 2017 [153], Copyright 2017 The Royal Society of Chemistry and reproduced with permission).

**Figure 9:**
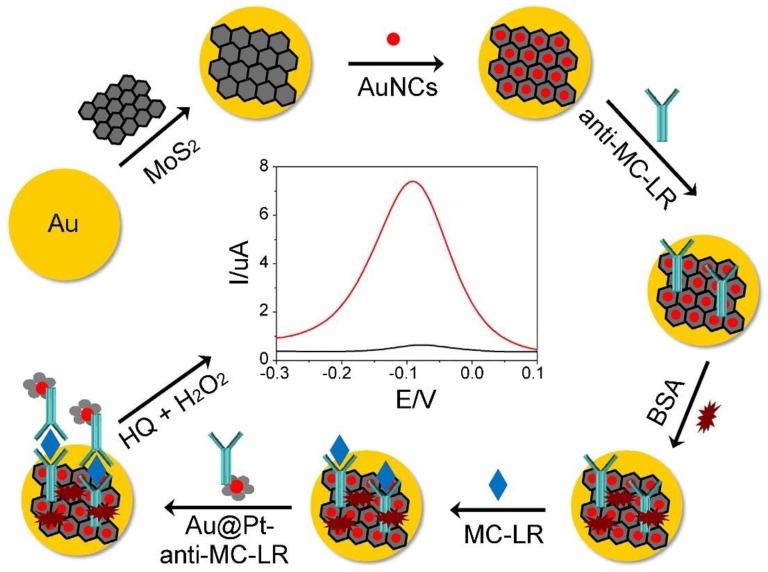
Schematic representation of the preparation and detection principle of the MC-LR immunosensor (adapted from Pang et al. 2018 [155], Copyright 2018 Elsevier B.V. and reproduced with permission).

Microcystins (MCs), a group of toxins produced by a number of cyanobacteria species, are monocyclic heptapeptides with the general structure cyclo(D)-Ala-X-(D)-erythro-b-methyl-iso-Asp-Y-Adda-(D)-iso-Glu-N-meth-yldehydro-Ala (X and Y represent L-amino acids). They are the most common cyano-toxins [138,139,140,141,142]. The unusual Adda amino acid, unique to MCs, is responsible for the toxicity of the molecule. There are more than 100 known variants of MCs, which are found in a wide variety of aquatic environments, in particular, eutrophic waters. Exposure to MCs via consumption of poisoned drinking-water or eating contaminated fish can cause permanent multiple organ injuries, developmental effects, reproductive effects and cancer. Therefore, it is important to develop highly sensitive methods for on-site monitoring of MCs. In addition, as the most potent congener, the Microcystin-LR (MC-LR) is commonly used to evaluate the toxicological data on the effects of MCs. The maximum tolerance limit of MC-LR concentration is 1 μg L^−1^ in different water sources by the WHO provisional guideline. Electrochemical biosensors, including 2D nanomaterial-based amperometric immunosensors, impedimetric aptasensors, and PEC aptasensors, have been extensively employed to detect MCs/MC-LR [143,144,145,146,147,148,149,150,151,152,153,154,155,156,157,158,159]. Li et al. have fabricated an electrochemical immunosensor based on GO-AuNP nanocomposites for MC-LR detection in water samples though layer-by-layer alternate electrodeposition of GO and chloroauric acid (HAuCl_4_) on the GCE surface for 20 cycles [147]. The GO-AuNP-decorated GCE was then modified by the conducting polymer (poly(2,5-di-(2-thienyl)-1-pyrrole-1-(p-benzoicacid)) and 1-iso-butyl-3-methylimidazolium bis(tri-fluoromethane-sulfonyl) imide ionic liquid (IL). A polyclonal antibody of MC-LR was immobilized on the electrode by the conventional EDC/NHS reaction. The GO-AuNP nanocomposites enhance electron transfer of Fe(CN)_6_^3^^−/4−^ to the electrode while the IL acts as the stabilizer of the antibody. The as-developed electrochemical immunosensor has good repeatability (e.g., RSD = 1.2%) and long-term stability (e.g., retain 95% activity over a 20 weeks storage period), and can detect MC-LR in water samples with a very low LOD of 3.7 × 10^−17^ mol L^−1^. Recently, He et al. synthesized a kind of magnetic rGO nanocomposite (Fe_3_O_4_@PDA/RGO) for constructing a MC-LR electrochemical immunosensor by using the hydrothermal treatment of Fe_3_O_4_ nanocluster@Polydopamine core@shell nanoparticles (Fe_3_O_4_@PDA) with GO (as shown in Figure 8) [153]. Due to its surface area and easy separation, the Fe_3_O_4_@PDA/RGO clearly enhances the antigen immobilization ability of the electrode. Then, a secondary-antibody and circularization DNA template were conjugated on gold nanorods (AuNRs) for recognizing the captured MC-LR-antibody pair on the Fe_3_O_4_@PDA/RGO-modified electrode surface and rolling circle amplification. Because the rolling circle amplification strategy can generate massive repeated DNA sequences, the signal of the immunosensor is greatly enhanced by hybridization of electrochemical active probes with the repeated DNA sequences. Under the optimal conditions, the as-developed immunosensor has good detection performance including a wide linear range (from 0.01 mg L^−1^ to 50 mg L^−1^) and a low LOD (0.007 mg L^−1^), which can be employed to detect MC-LR in real samples (e.g., river water). A series of PEC aptasensor-based various GO/rGO nanocomposites have been developed for sensitively detecting MC-LR since the PEC method has been considered to be a more sensitive technique, ascribed to the combination of electrochemical and optical techniques [149,151,157]. For instance, Du et al. developed a PEC aptasensing platform based on AgI-nitrogen-doped graphene (AgI-NG) nanocomposites as photo-cathodes and a MC-LR aptamer as the recognition unit [157]. The PEC aptasensor has a LOD of 3.7 × 10^−17^ mol L^−1^, which can be employed to determine MC-LR in inaquatic products (e.g., fish extracts). As a graphene analogue, the MoS_2_ nanosheet is also expected to serve as an excellent functional material for development of electrochemical biosensors. As shown in Figure 9, Pang et al. constructed an enzyme-free electrochemical immunosensor for detecting MC-LR based on a unique competitive detection scheme using MoS_2_ nanosheets/BSA-stabilized gold nanocluster (MoS_2_/AuNCs) nanocomposites and Au core/Pt shell nanoparticles (Au@PtNPs) [155]. Due to its large surface area and excellent biocompatibility, the MoS_2_/AuNCs nanocomposite was employed as a platform for improving the biological activity and immobilizing amount of antibody on the electrode surface. The as-developed enzyme-free electrochemical immunosensor has good stability (e.g., 92% of the initial level remained after being stored at 4 °C for four weeks), and exhibits a wide linear range of 1.0 ng L^−1^–1.0 mg L^−1^ with a LOD of 0.3 ng L^−1^. The practicability of the as-developed immunosensor has been demonstrated by detection of MC-LR in various water samples including tap water, lake water, and river water. The MC-LR amounts in these water samples detected by the immunosensor are consistent with those determined by the conventional ELISA method. Very recently, Liu et al. developed an electrochemical aptasensor for sensitive and selective determination of microcystin-LR by using a dual signal amplification system consisting of a ternary nanocomposite and HRP [159]. The ternary nanocomposites were prepared by depositing AuNPs on the MoS_2_ nanosheets covered with TiO_2_ nanobeads (TiONBs). The MoS_2_ nanosheet-modified TiONBs provide a large surface area for efficiently immobilizing AuNPs and thiolated MC-LR aptamers. Due to the combination of good electron transfer and high catalytic capability of the ternary composite, the aptasensor has a wide dynamic range from 0.005 to 30 nmol L^−1^ and a LOD of 0.002 nmol L^−1^.

### 4.2. Cylindrospermopsin

**Figure 10:**
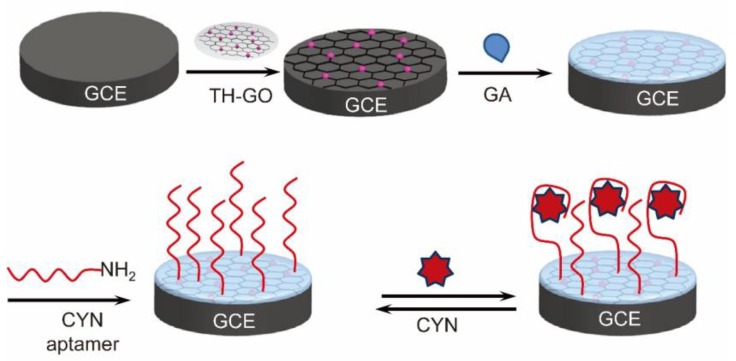
Schematic representation of the label-free impedimetric aptasensor for detecting cylindrospermopsin (adapted from Zhao et al. 2015 [167], Copyright 2015 The Royal Society of Chemistry and reproduced with permission).

Cylindrospermopsin (CYN), a tricyclic alkaloid with a molecular mass of 415 Da, is a common cyanotoxin, and is produced by cyanobacteria including *Cylindrospermopsis*, *Anabaena*, *Umezakia,* and *Aphanizomenon* [160,161,162,163,164,165,166]. Cylindrospermopsin can cause DNA/RNA strand breakage and promote hepatotoxicity, cytoxicity, and genotoxicity through inhibiting protein translation and binding to DNA. The Falconer recommends a tentative guideline value of 1 ug L^−1^ for cylindrospermopsin [166]. Recently, we fabricated a label-free impedimetric aptasensor based on a GO-thionine (TH-GO) nanocomposite for detection of CYN by covalent binding of the amino-terminated aptamer of CYN to TH-GO nanocomposite-modified GCE via glutaraldehyde (as shown in Figure 10) [167]. Using [Fe(CN)_6_]^4−/3−^ as an electrochemically active probe, CYN can be detected as low as 0.117 ng mL^−1^ in water. The as-proposed aptasensor has been employed for detecting CYN in spiked lake water samples, and satisfactory recoveries were obtained. With its superior performance characteristics combined with long-term stability (it retained approximately 74.7% of its initial value after being stored at 4 °C for 30 days) and excellent reusability (RSD = 2.1% within 10 reacting cycles), the as-developed aptasensor is a potential candidate for on-site CYN analysis.

### 4.3. Saxitoxins

As a group of carbamate alkaloid neurotoxins, saxitoxins (STXs) contain sixteen variants, which are commonly associated with “red tides,” and found as a paralytic shellfish toxin. Australia has a drinking water guideline of 3 μg L^−1^ of STX equivalence. Recently, Bratakou et al. constructed a miniaturized potentiometric STX immunosensor on graphene nanosheets with incorporated lipid films and anti-STX (the natural STX receptor) [168]. The potentiometric STX immunosensor can be easily miniaturized because graphene nanosheets have a high surface area and good conductivity, and incorporate well with the lipid bilayer membrane for immobilizing anti-STX antibody. The potentiometric STX immunosensor exhibits several advantages such as a rapid response time (ca. 5–20 min), low LOD (1 nmol L^−1^) with high sensitivity (ca. 60 mV/decade of toxin concentration), good reproducibility (maximum deviation only 6.8%), reusability, high selectivity and long shelf life (> 1 month). The practicability of the method was demonstrated by detecting STX in lake water and shellfish samples. This graphene nanosheets with incorporated lipid films could be used to develop biosensors for monitoring other toxins.

### 4.4. Brevetoxin B

Brevetoxins (BTXs) are potent cyclic polyether neurotoxins, which are naturally produced by the marine “red tide” dinoflagellate, *Karenia brevis*. BTX exposure can cause neurological shellfish poisoning (NSP), which has increased in geographical distribution over the past decade [139]. As early as 2012, Tang et al. constructed a magneto-controlled electrochemical immunosensor for sensitive detection of brevetoxin B (BTX-2) in seafood by using guanine-assembled graphene nanoribbons (GGNRs) as molecular tags on a home-made magnetic carbon paste electrode [169]. In this case, the GGNRs were modified by bioconjugates of BSA with BTX-2 (BTX-2-BSA), while monoclonal mouse anti-BTX-2 antibodies were covalently immobilized on the surface of magnetic beads for the capture of BTX-2 through a competitive-type immunoassay format. The formed magnetic immunocomplex was integrated on the electrode with an external magnet, followed by determination in pH 6.5 phosphate-buffered solution containing 2 μmol L^−1^ Ru(bpy)_3_Cl_2_. Compared with pure guanine-labeled molecular tags, the GNR-labeled electrochemical immunoassays show a much wider linear range and lower detection limit. Under optimal conditions, the electrochemical signals decreased by increasing concentration of BTX-2 in the sample. The magneto-controlled immunosensing platform has a wide dynamic range from 1.0 pg mL^−1^ to 10 ng mL^−1^ with a LOD of 1.0 pg mL^−1^ BTX-2. The analytical reliability of the magneto-controlled electrochemical immunosensing platform is demonstrated by the detection of BTX-2 in 12 spiked samples including *S. constricta, M. senhousia and T. granosa*. The as-obtained results are consistent with those of traditional ELISA.

### 4.5. Okadaic Acid

**Figure 11:**
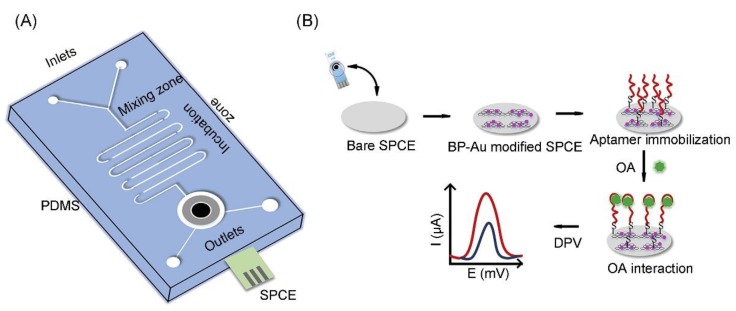
**A** microfluidic electrochemical aptasensor for the detection of okadaic acid: (**A**) graphic of the fabricated PDMS microfluidic chip, and (**B**) schematic representation of the process of aptamer-based sensing (adapted from Ramalingam et al. 2019 [172], Copyright 2019 Elsevier B.V. and reproduced with permission).

The family of okadaic acid (OA) biotoxins consists of OA and its analogues dinophysistoxins 1, 2 and 3 (named as DTX-1, DTX-2 and DTX-3) [170]. As a by-product of harmful algal blooms (HABs), OA originates from the algal genera *Prorocentrum* and *Dynophysis.* Eissa and Zourob developed a direct competitive voltammetric immunosensor for the sensitive detection of OA based on carboxyphenyl-functionalized graphene-modified SPCEs (GSPCEs) [171]. The anti-OA antibodies were immobilized on the GSPE via carbodiimide chemistry, where OA and OA-ovalbumin (OA-OVA) in solution compete for their binding to the immobilized antibody. Benefitting from the unique electrochemical properties of graphene and the stability of the carboxyphenyl layer, the immunosensor exhibits a linear response up to 5000 ng L^−1^ with a LOD of 19 pg mL^−1^. The immunosensor was successfully applied for detecting OA in the spiked shellfish extracts, showing good recovery. Very recently, Ramalingam et al. fabricated an electrochemical microfluidic biochip for detecting OA by using phosphorene-gold (BP-Au) nanocomposite-modified SPCE (as shown in Figure 11) [172]. The as-synthesized BP-Au nanocomposite not only serves as a backbone to the aptamer sequence, but also significantly enhances the electrochemical response of the aptasensor. DPV measurements revealed a LOD of 8 pmol L^−1^, while a linear range was found between 10 nmol L^−1^ to 250 nmol L^−1^. The electrochemical aptasensor has excellent selectivity and can be employed to detect OA in fresh mussel extracts. The results suggest that the microfluidic electrochemical aptasensor can be served as an easy-to-use POC device for an on-field assay.

## 5. Conclusions and Perspective

This review has summarized the recent progress in electrochemical biosensing systems for the determination of various microbial toxins by using 2D nanomaterials and their nanocomposites (hereinafter referred to 2D nanomaterials). The literature results demonstrate that the integration of 2D nanomaterials into electrochemical biosensors has led to the significant enhancement of their analytical efficiency, including a high sensitivity (e.g., very low LODs) with a wide linearity range over several orders of magnitude, rapid assaying time, and simplified analytical procedures, and they are also suitable for on-site monitoring. During the determination processes, 2D nanomaterials mainly have two roles: as substrates for efficient immobilization of capturing biomolecules (e.g., anti-toxin antibodies and aptamers) and high active electrochemical probes for signal amplification. Some 2D nanomaterials have multifunctionality, and are capable of playing both of the above roles. Furthermore, the 2D nanomaterial-based electrochemical aptasensors have been proven as reusable platforms for detecting toxins.

Although the 2D nanomaterial-based electrochemical biosensors show great promise within laboratory investigations, such as the detection of toxins in buffer solutions and/or toxin-spiked samples, the technique remains relatively immature in development compared with standard toxin assaying tools (e.g., HPLC and ELISA), and several technical challenges are still awaiting further investigation. (1) The multiple electrode modification steps are normally required for increasing the recognition performance of the immobilized aptamer or antibody, and reducing background signals. This phenomenon requires manual and tedious work, which not only increases the preparation cost of biosensors, but also leads to poor reproducibility of the results among laboratories. In order to simplify the biosensor construction procedure, future research should increase the reaction efficiency of 2D nanomaterials with biomolecules (such as an antibody and apatmer) and decrease unreacted activity groups on the surface of 2D nanomaterials after biomolecule immobilization. Furthermore, development of automatic methods for modification of 2D nanomaterials on the electrode surface may help to increase the inter-laboratory reproducibility of biosensors. (2) The properties of 2D nanomaterials, including their electrical conductivity, PEC conversion capability and biomolecule immobilization capacity, are strongly dependent on their morphology, such as shape, size, purity, and defects. Therefore, 2D-nanomaterials should be fully characterized before biosensor fabrication. In further research, researchers are strongly encouraged to establish the synthesis standard of 2D-nanomaterials in order to improve the reproducibility of 2D nanomaterial-based electrochemical biosensors. In addition, the as-proposed synthesis strategy should be easily employed to produce 2D-nanomaterials on a large-scale by simply adjusting the synthesis conditions, such as increasing the amount of reactants. This factor is very important for industrialization of the 2D nanomaterial-based electrochemical sensors. (3) To date, one kind of 2D nanomaterial-based electrochemical biosensor is merely confined to determine a single microbial toxin. Because of coexistence of various microbial toxins in nature, future research should focus on development of a universal biosensor production technology for enabling rapid analysis of various toxins. (4) In order to achieve large-scale application, in particular for on-site monitoring, further efforts should be directed toward the development of 2D nanomaterial-based electrochemical biosensors, which can be used to detect toxins in practical samples such as various agricultural, food stuff, body fluids, and environmental sectors (e.g., lake water and sea water). The practicability of 2D nanomaterial-based electrochemical biosensors could be improved through integration of the biosensor with other techniques such as microfluidic devices and microarrays because miniaturization will help to increase the detection throughput, e.g., recognize multiple elements simultaneously. (5) Currently, aptamers and antibodies are mainly used for recognition of the toxins. In order to obtain high selectivity, the key epitope residues of the aptamer and antibody should be unrestrained after immobilization on the 2D nanomaterials. In addition, the molecular structures of the aptamer and antibody are sensitive to the environmental conditions (such as temperature, ionic strength and interferences from sample matrices). The high apparent affinity of the aptamer and/or antibody could be achieved through immobilization of the aptamer and/or antibody by stereoselective reactions (e.g., chick chemistry, DNA hybridization, biotin-avidin recognition). In addition, future research should aim to increase the biocompatibility of 2D nanomaterials. Finally, we expect commercialization of 2D nanomaterial-based electrochemical biosensors into practical procedures for detecting multiple toxins in practical samples through efforts of researchers in different disciplines, which would give significant benefit to the public.
